# Stigmatising and Racialising COVID-19: Asian People’s Experience in New Zealand

**DOI:** 10.1007/s40615-022-01448-7

**Published:** 2022-11-11

**Authors:** Liangni Sally Liu, Xiaoyun Jia, Andrew Zhu, Guanyu Jason Ran, Richard Siegert, Nigel French, David Johnston

**Affiliations:** 1 School of Humanities, Media and Creative Communication, Massey University, Auckland, New Zealand; 2 Institute of Governance & School of Politics and Public Administration, Shangdong University, Qingdao, China; 3 School of Mathematical and Computational Sciences, Massey University, Auckland, New Zealand; 4 Trace Research Ltd, Auckland, New Zealand; 5 School of Applied Sciences, Edinburgh Napier University, Edinburgh, UK; 6 Department of Psychology & Neuroscience, School of Clinical Sciences, University of Technology, Auckland, New Zealand; 7 Infectious Diseases Research Centre, Hopkirk Research Institute, Massey University, Palmerston North, New Zealand; 8 Joint Centre for Disaster Research, Massey University, Wellington, New Zealand

**Keywords:** Asian people, COVID-19, Racial discrimination, Stigmatisation, New Zealand

## Abstract

The Asian community — the second largest non-European ethnic community in New Zealand — plays an important role in combatting the COVID-19 pandemic, evidenced by their active advocation for border control and mass masking. Despite the long history of racial discrimination against the Asian population, the Asian community has experienced certain degrees of racial discrimination associated with the stigmatisation as the cause of the COVID-19 outbreak in New Zealand. Based on data from a quantitative online survey with 402 valid responses within the Asian communities across New Zealand and the in-depth interviews with 19 Asian people in Auckland, New Zealand, this paper will illustrate Asian people’s experience of racial discrimination and stigmatisation during the pandemic in the country. The survey shows that since the outbreak of COVID-19, under a quarter of the participants reported experiencing discrimination, and a third reported knowing an immediate contact who had experienced discrimination. However, when looking beyond their immediate social circle, an even higher proportion reported noticing racism and stigmatisation through the traditional or social media due to COVID-19. Major variations of the degree of racial discrimination experienced are determined by three demographic variables: ethnicity, age, and region. The in-depth interviews largely echoed the survey findings and highlighted a strong correlation between the perceived racial discrimination among the local Asian community and the stigmatisation associated with COVID-19. These findings are important for improving the way we manage future pandemics and other disasters within the context of the UN Sendai Framework for Disaster Risk Reduction.

## Introduction

Since the outbreak of the COVID-19 pandemic, the world has witnessed how the COVID-19-related stigmatisation contributed to a series of racist, violent attacks, and bullying against Chinese and other Asians, especially in some Western countries [1–6]. What surrounds this phenomenon is the rising tension between China and some major Western powers. Such COVID-19-driven politics have stirred anti-China, and anti-Chinese and anti-Asian sentiment in the West, especially in the USA [7–10]. For example, the finger-pointing that implies COVID-19 originated in China has worsened China-USA relations which have been tense since 2008 [10]. Some Western leaders, such as the previous president of the USA, Donald Trump, queried the origins of the virus [11–14]. This scenario led not only to individual cases of bullying and violence towards people with Asian appearance but also to a global sentiment of Asian hatred [15]. Elsewhere, in Europe and Australia, such violence emerged in the early months of the global outbreak [15, 16].

The situation in New Zealand is comparatively better. Firstly, the New Zealand Government’s response to the pandemic is largely efficient and successful. Before the country started to advocate mass vaccination in mid-2021, the government embraced an elimination strategy to contain the virus spread and accordingly enforced very restrictive measures to combat the outbreak. This includes implementing strict border controls, compulsory Managed Isolation and Quarantine (MIQ) for international arrivals from overseas, two nationwide lockdowns when the widespread community transmission of the virus was detected, the encouragement of extensive testing [17, 18], and the advocation for high uptake of the vaccination since it became available in early 2021 [19]. Consequently, New Zealand seemed to respond to the pandemic efficiently and won an international reputation as its measures to combat the pandemic are some of the toughest in the Western world [20–23].

Secondly, unlike some other Western countries where deep-rooted racial discrimination and stigmatisation towards certain ethnic groups of Asian appearance were catalysed by their governments’ COVID-19 rhetoric and mainstream media’s racist sentiment and comments [11, 14–16], New Zealand’s handling of ethnic and racial relations during the pandemic was much more sensible and humane. The government’s slogan ‘Unite Against COVID-19’ and ‘Team of Five Million’ underlines the importance of solidarity across races and ethnicities from the beginning of its pandemic response [18]. Also, anti-racist government leadership was advocated through dedicated governmental agencies [24]. The New Zealand mainstream media engaged in very few anti-Chinese stigmatisation narratives in its reporting, as evidenced that the terms ‘Chinese virus’ and ‘China virus’ that were widely used by some conservative news media at the onset of the outbreak in other Western countries cannot be found in New Zealand mainstream media. Under this relatively friendly social environment, the local Asian community — the second largest non-European ethnic community in New Zealand — has played an important role in leading the combat with the COVID-19 pandemic, evidenced by their active advocation for border control and mass masking [18].

However, this does not mean that pandemic-related discrimination and stigmatisation do not exist in this country. New Zealand Human Rights Commission has received increasing numbers of complaints from the Chinese and other Asian communities about COVID-19-fuelled racist and xenophobic abuse since the virus outbreak in early 2020. They reported that the racial abuse they experienced was closely associated with the stigmatisation as the blame for the cause of the COVID-19 outbreak [25]. The ‘Stop Asian Hate’ rally protest in Auckland on 27 March 2021 was a response to such an intensified racial relation and aimed to raise public awareness and call for stopping discriminations against Asian people [26].

Based on a quantitative online survey with 402 valid responses from the local Asian community of New Zealand and in-depth interviews with 19 Asians in Auckland, New Zealand, this paper casts a light on the Asian people^1^’s individual experiences of racial discrimination at the interpersonal level and stigmatisation in the pandemic context of New Zealand in 2020. It shows to what extent have Asian people experienced pandemic-related racial discrimination and stigmatisation and whether the perceived racial discrimination and stigmatisation associated with COVID-19 is correlated. It also examines possible associated factors that have caused racial discrimination and stigmatisation. Overall, the paper provides a discussion on the intersectionality of the COVID-19-related racial stigmatisation and discrimination, health governance, and global pandemic politics.

## Stigmatisation and Prejudice/Discrimination in Health Research

Stigma and prejudice/discrimination have been important focuses of research for decades, and these two research traditions are closely related to each other. For example, stigma and prejudice/discrimination are often conceptualised as negative attitudes held by individuals or groups who carry unfair perceptions towards other specific groups of people [27]. Both also emphasise structural and interpersonal experiences of unfair treatment and violence perpetrated against people who belong to vulnerable social groups [27, 28]. In other words, these two research traditions share a conceptual similarity. However, they are from disparate disciplinary origins, which determines that the subjects of their research interests and focuses are quite different [28].

Research on stigma has principally originated from social psychology [29] and has mainly focused on studying people with special health conditions or social behaviours, for example, people with mental illness or HIV [30]. In contrast, research on prejudice/discrimination is mainly derived from the discipline of sociology. It largely focuses on far more general populations, but their social characteristics, such as gender, age, race, and class divisions, have powerful implications on their positioning in the social, economic, and political hierarchy. In this sense, discrimination can extend to different domains and there are different types of discrimination, such as racial, gender, age, and sexual orientation discrimination [31]. In the USA context, this research tradition focuses on growing social concerns of racism, especially on race and ethnicity as determinants for different social consequences and how racial discrimination is driven by prejudice historically [27, 31]. As an illustration, Stuber et al. pointed out that the research tradition of prejudice/discrimination grew from concerns with social processes driven by social inequity, such as exploitation and domination based on race, while research on stigma has been more concerned with processes driven by established social normality and disease avoidance [28]. In addition, there are different levels of racial discrimination, including interpersonal, organisational, structural, and institutional racial discrimination [31–33]. While interpersonal racial discrimination refers to individual behaviours or violence towards non-dominant racial groups, institutionalised processes of racial discrimination, namely, institutional or interchangeably structural racial discrimination, take place when valued opportunities and resources are structured by race, which then results in a broader ideological and policy environment that empowers dominant group by disproportionally allocating desirable societal opportunities and resources to non-dominant racial groups. Both forms of racial discrimination can have negative effects on health [32, 33].

More recently, there has been an emergence of research interests that focus on understanding the linkages between stigma and racial prejudice/racial discrimination and health. In this research spectrum, the two disparate concepts of stigma and racial prejudice/racial discrimination are framed as important contributors to the negative health outcomes of some racialised non-dominant groups [35–37]. In Meyer’s research [38], both stigma and racial prejudice/racial discrimination have been examined in relation to so-called minority stress to address the negative psychological and mental health outcomes of racialised ethnic minorities. Under such a framework, both stigma and prejudice/racial discrimination are psychosocial stressors in the lives of racialised non-dominant groups but function in different ways in impacting their health outcomes negatively. In detail, the negative health impact of prejudice/discrimination is through interpersonal interactions where dominant people mistreat non-dominant people [36–39], while the negative health impact of stigma is often through an internalised belief and anxiety of the anticipation of negative treatment by members of dominant groups. This is named as internalised racism — a major psychological effect of racism in which ‘the individual inculcation of the racist stereotypes, values, images, and ideologies perpetuated by the White dominant society about one’s racial group, leading to feelings of self-doubt, disgust, and disrespect for one’s race and/or oneself’ [40: 553]. This means that the form of stress caused by stigma can exist and/or persist even when unfair treatment is not experienced [34–36].

In the context of research on contagious diseases, the two research traditions have been re-conceptualised as having a close relationship. In detail, the perceived association between a contagious disease and certain ethnic group(s) or place(s)/country(ies) is frequently a cause of discriminative behaviours and actions towards those ethnic groups [27, 28]. Quite often, stigmatised foreigners and migrants are perceived as the origin or potential carriers of a contagious disease and to pose a health threat to the public; and therefore, they frequently experience unfair treatment or even become the target of racist discrimination and violence [37–39]. Historical evidence shows the crucial impact of the contagious diseases associated stigmatisation on shaping societal-level reactions towards foreign nationals. The underlying psychological driver is the fear of the disease and feeling the need to blame or shame certain marginalised groups of people for the cause of infectious diseases. For instance, the homophobia that characterised much of the early public response to the HIV/AIDs epidemic [35] and the blame discourses against the Chinese during the 2003 SARS epidemic outbreak in the USA [41, 42]. From a psychological perspective, stigmatisation is usually manifested through the so-called disease-avoidance behaviours [43, 44]. Consequently, such practices are often the cause of people’s feeling of being the target of racial discrimination. A systematic literature review completed by Yashadhana et al. in particular points out that pandemic-related racism had subsequent negative impacts on mental health and health care accessibility of racially minoritised people [45].

In modern society, when new media becomes viable, it often becomes the platform for disease-related xenophobic propaganda in which ethnic labelling of a contagious disease is used in the risk discourse, thus, resulting in ‘disease-avoidance’ behaviours and even hostile attitudes towards certain ethnic groups [41, 46, 47]. The paper uses the theories discussed above as an analytical lens to interpret the findings from the empirical research.

## Research Design and Methods: a Mixed-Method Research

The research reported in the present paper adopted a mixed-method research design involving qualitative and quantitative methods to collect empirical data. The quantitative component of the research is based on an online quantitative questionnaire survey conducted in the late 2020 among the Asian community of New Zealand. The qualitative component of the research comprises one-to-one in-depth interviews with 19 Asian people completed in Auckland between November 2020 and July 2021. Data from both quantitative and qualitative methods are then complementarily analysed towards answering the research questions. While the survey provides quantitative evidence of the extent of racial discrimination that the local Asian community has experienced under the pandemic context, the data from the in-depth interviews offers rich insights towards understanding the causes of racial discrimination and stigmatisation. Theoretically, using both quantitative and qualitative methods for the same subject may produce a conceptual triangulation strategy offsetting or counteracting any biases generated from each of these methods [48, 49]; thus, improving the research quality.

### Detailed Methods Used for the Quantitative Survey

Employing the 2018 New Zealand Census’s Asian adult population distribution by age, gender, and location, the stratified sampling technique was used in the online survey to ensure the representativeness of all Asian New Zealanders in the country. The researched subject — Asian people — were identified by their countries of origin. Both overseas-born and New Zealand local-born Asians were included in the sampling, although the local-born Asian people comprised a small percentage of the full sample. This survey was contracted out to an external research company to conduct. Data was independently collected by the company between the 5th and 18th of December 2020. The ethnic Chinese sample was collected through the company’s Chinese Immigrants Research Panel. All other Asian ethnic samples were collected from the company’s partner online panel (by random email invitations of *n* = 1101, the total response rate is 36.5% when all quotas were filled). Only complete questionnaires without missing answers could be submitted for analysis. Each respondent was allowed to submit the questionnaire once only, according to the IP address recorded by the research panels.

The survey obtained 402 valid responses. Among these valid responses, 49% were from males, 50.5% were females, and two respondents stated their gender as other and were excluded from gender differences analyses because the number is too small to satisfy the requirements of conducting *chi*-square tests. Respondents were all over 18 years old, originally from more than 14 Asian countries/particular areas and spread across 17 regions in New Zealand. The overall margin of error is ± 4% at the 95% confidence level. The sociodemographic data captured in this survey included gender, country of origin (a proxy for ethnicity), place of residency, and age. A complete sample composition breakdown is provided in Table 1.

**Table 1:** The sample composition breakdown

	(%) Sample distribution	(*n*) Sample size
Gender		
Male	49.0%	197
Female	50.5%	203
Other	0.5%	2
Country of origin*		
Mainland China	31.3%	126
Hong Kong	2.0%	8
Taiwan	1.3%	5
Vietnam	1.5%	6
Cambodia	1.3%	5
India	33.6%	135
The Philippines	10.3%	41
Korea	5.0%	20
Japan	2.5%	10
Sri Lanka	2.3%	9
Singapore	2.1%	8
Malaysia	4.7%	19
Thailand	0.9%	4
Other Asian countries	1.3%	5
Age groups		
18–29 years	33.4%	134
30–49 years	35.1%	141
50–64 years	23.4%	94
65 years +	8.1%	33
Region		
Northland Region	1.6%	6
Auckland Region	65.1%	262
Waikato Region — Hamilton	3.8%	15
Waikato Region — other	0.2%	1
Bay of Plenty Region	1.6%	6
Hawke’s Bay Region	0.7%	3
Taranaki Region	1.1%	4
Manawatu-Wanganui Region	2.2%	9
Wellington Region	12.8%	52
Tasman Region	0.3%	1
Marlborough Region	0.5%	2
Canterbury Region — Christchurch	7.0%	28
Canterbury Region — other	0.5%	2
Otago Region — Dunedin	0.8%	3
Otago Region — Queenstown	0.5%	2
Otago Region — other	0.3%	1
Southland Region	1.0%	4
Total	100%	402

(Note: *The sample includes 31 New Zealand-born Asians)

The analysis of the quantitative data is twofold. The first part is a *chi*-square test of discrimination and stigmatisation by demographic variables. The second part is a correlation analysis to investigate the hypothesised correlation between the perceived discrimination and noticed stigmatisation of COVID-19 in the research. To measure the existence and level of pandemic-driven racism, discrimination, and stigmatisation, four questions were asked based on the distance of the respondents’ social ties, ranging from oneself to significant others, to media environment, and the public perception in general. These four questions are: (1) Since the COVID-19 outbreak in New Zealand, have you been discriminated against (e.g. making offensive remarks about your race, verbal/physical abuse) because of your ethnicity? (2) Since the COVID-19 outbreak in New Zealand, have you known people in your immediate social environment who had encountered racist comments and/or discrimination against their ethnicity? (3) Since the COVID-19 outbreak in New Zealand, have you noticed any racist comments related to the pandemic against your ethnicity in the media/social media? And (4) have you noticed any race-related stigmatisation about COVID-19 during the pandemic? Each question was measured on three items: ‘yes’, ‘no’, and ‘not sure’, and frequency analyses were performed. The Pearson *chi*-square tests were used to test independence among the respondents for four key demographic differences, including gender, ethnicity, age, and region. A *p*-value of less than 0.05 was considered statistically significant. The analyses were performed using SPSS.

The second part of analysing the quantitative survey data is a Pearson correlation analysis to investigate the hypothesised correlation between the perceived discrimination and noticed stigmatisation of COVID-19 in the research. As discussed before, the theoretical rationale is that stigmatisation and racial discrimination are closely related to each other in a pandemic context where when people fear a transmissive disease, they tend to stigmatise certain group(s) of people by blaming them as the cause of the disease. This situation might result in aggressive discriminatory behaviours towards those stigmatised group(s). Based on this theoretical hypothesis, the framed hypothesis for this research is that the perceived stigmatisation of the pandemic associated with China, Chinese, and Asian-looking people will highly likely lead to an increasing sense of being racially discriminated against by these people. Therefore, two survey questions are chosen to form this correlation analysis. They are (1) Have you been discriminated against because of your ethnicity? and (2) Have you noticed any stigmatisation associated with COVID-19 during the pandemic? Each question was measured on three items (‘yes’, ‘no’, and ‘not sure’). The answer ‘not sure’ was not included in the analysis since their counts are limited. Correlation coefficient (*r*) was used to measure the strength of the relationship between two variables. The correlation coefficient should be between − 1 and 1. The absolute value of the correlation coefficient indicates the relationship strength, namely, the larger the number, the stronger the relationship. In general, a value between 0.3 and 0.5 is considered a moderately strong correlation between two variables, a value above 0.5 is considered a strong correlation, and a value below 0.3 is considered a weak correlation [50].

### Detailed Methods Used for the Qualitative Interviews

In total, 19 in-depth one-on-one interviews with recruited adult Asian people were conducted in Auckland from November 2020 to July 2021 either via ZOOM or face-to-face. Purposive sampling was carried out in the beginning based on the authors’ existing social networks within the New Zealand Asian community. After that, the snowballing technique was applied to reach more participants. We found that the data collected began to saturate after interviewing 19 Asian people. Moreover, given the nature of qualitative research which is not seeking generalisation like quantitative research, the open-ended interview questions were designed to draw in-depth insights from the participants to answer the research questions. All participants were over 18 years old, originally from Asian countries, and had obtained New Zealand residency, permanent residency, or citizenship. Table 2 shows that our participants are from various Asian countries, including Mainland China, Malaysia, India, and the Philippines. Each interview lasted from 40 to 90 min. The language used in the interviews was either Mandarin or English upon the interviewees’ choice. The interview questions covered their interpersonal experiences and perceptions of pandemic-related racial discrimination and stigmatisation (i.e. Have you observed any racism or racial attack during the pandemic? What is your general opinion about racism in the New Zealand context? What do you think about the use of the term ‘Chinese virus’?). These interview questions aim to explore the participants’ lived experience of the pandemic-related racial discrimination, perceived stigmatisation of the pandemic, and reasoning of such experience and perceptions; thus, towards understanding the factors that have contributed to the perceived stigmatisation of the pandemic and caused people to feel that they had been racially discriminated.

**Table 2:** Interviewee profile

Total interviewees	Gender	Age	Ethnicity	Region
*n* = 18	Female: *n* = 5 Male: *n* = 14	18–29 years: *n* = 2 30–49 years: *n* = 11 50–64 years: *n* = 4 65 years plus: *n* = 1	Chinese: *n* = 14 Indian: *n* = 3 Filipinos: *n* = 1 Malaysian: *n* = 1	Auckland: *n* = 19

All interviews were conducted by two Asian members of our research team and audio-recorded and later transcribed for analysis. The Asian ethnic background of the two researchers provided advantages to progress the research. As being a part of the Asian community, this positionality offered the researchers a unique opportunity to build social bridges with their interviewees. It was the cultural similarities between these interviewees and the two researchers that led to a more comfortable interview environment and open interview conversations. In addition, the shared experiences as Asians in the pandemic context gave the researchers an opportunity to effectively interpret the experience.

Thematic analysis was performed on the interview transcripts. Firstly, two transcripts were chosen for the two researchers to read closely to identify themes separately to form their own coding schemes. Secondly, the two researchers shared and discussed their coding schemes for internal validation, and then worked together to refine and unify the coding scheme. Based on the finalised coding scheme, one researcher completed the coding, and another checked. Finally, all codes were systematically reviewed and re-organised into different themes and sub-themes. Using thematic analysis enabled us to detect common patterns of interviewees’ answers.

## Results 1: Quantifying the Discrimination and Stigmatisation Experience — What Does the Survey Tell Us?

### Chi-Square Test of Discrimination and Stigmatisation by Demographic Variables

**Fig. 1:**
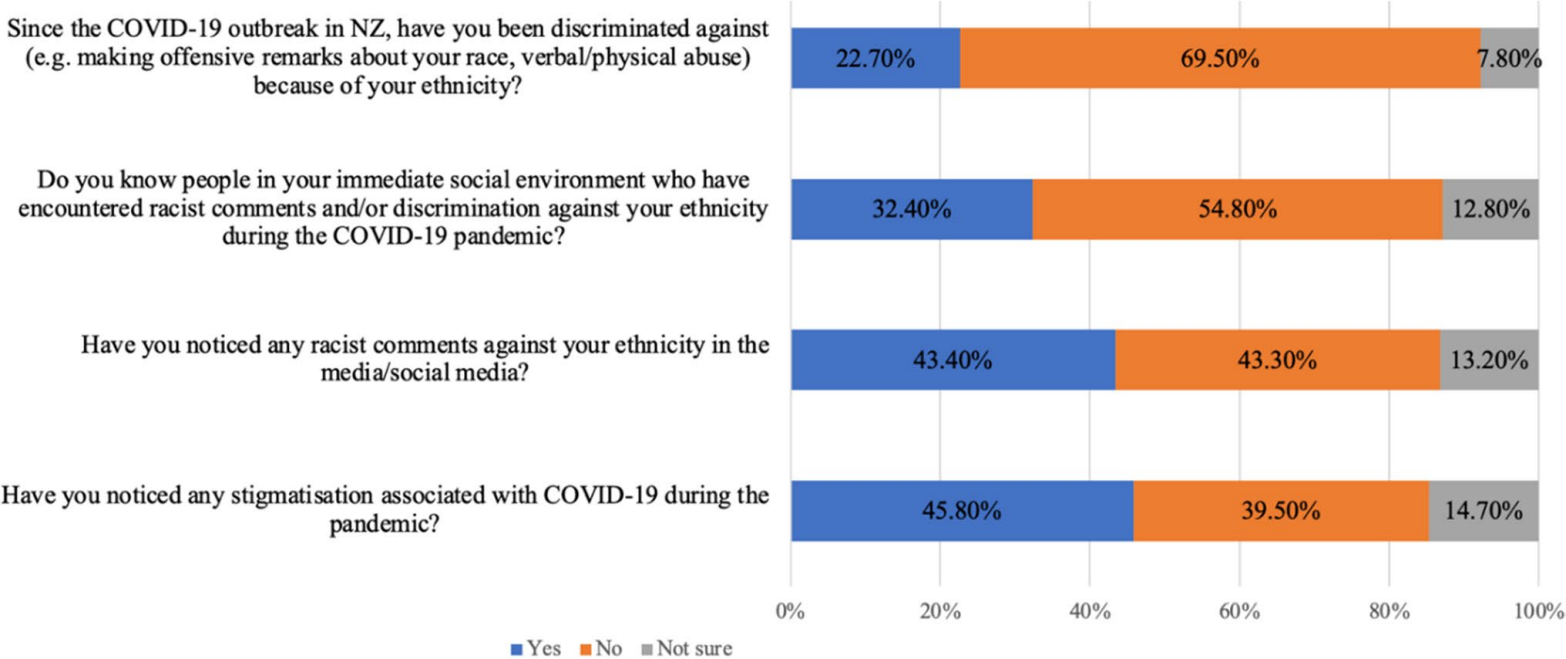
Asian’s experience of pandemic-related racial discrimination and stigmatisation

This survey firstly found that just under a quarter of the Asian respondents reported experiencing discrimination (22.7%), and a third reported knowing an immediate contact who had experienced discrimination (32.4%). However, when looking beyond their immediate social circle to ask their observation of comments in the media/social media, an even higher proportion reported noticing racism (43.4%) and stigmatisation (45.8%). The details of the percentage of each question are shown in Fig. 1. Overall, personal experience of racism or discrimination is lower in the spectrum but still significant. However, when the broader environment is considered, racism and discrimination encountered and observed is higher and likely influenced by media and events outside of New Zealand. If translating the percentage of direct discrimination experienced by the Asian respondents (27.3% of the total surveyed) into a population-based number based on the total Asian population of New Zealand (707,598) [51], that is around 193,174 adults with Asian ethnicities in the country who experienced pandemic-related racial discrimination. This number could be counted as serious.

It is worth noticing that the answer of ‘not sure’ was not considered in our analyses of survey questions 1, 2, and 4 because their counts of answers were limited, which does not satisfy the requirement of the *chi*-square test. For the same reason, ethnic groups were re-categorised into Chinese, Indians, and ‘other Asians including Filipinos’, and age groups over 50 years old were re-categorised into a single group of 50 years + for survey question 1 (see Table 3)*. Chi*-square tests show that ethnicity (*χ*^2^ = 12.026; df = 2; *p* = 0.002 < 0.05) and age (*χ*^2^ = 10.104; df = 2; *p* = 0.006 < 0.05) are two variables that have resulted in statistically significant differences in respondents’ answers about their personal experience of racism or discrimination. Table 3 shows that significantly more Chinese (35.4%) have perceived direct discrimination than Indians (18.1%) and ‘other Asians including Filipinos’ (20.9%). There are also significant differences in the ‘no’ answer to this question. Significantly, more Indians (81.9%) and ‘other Asians including Filipinos’ (79.1%) reported that they had not experienced discrimination than Chinese (64.6%). In addition, significantly more respondents aged 18–29 years (31%) and aged 30–49 years (27.5%) have perceived direct discrimination than those aged 50 and over (14.3%). Similarly, significantly more respondents who are 50 years old and above (85.7%) have reported that they did not experience any direct discrimination than those aged 18–29 years old (69%) and 30–49 years old (72.5%).

**Table 3:**
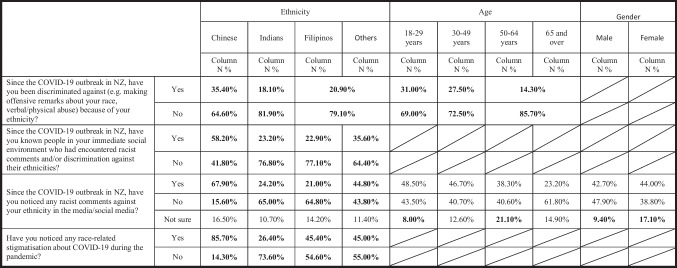
Respondents’ experience of the COVID-19-related racial discrimination and stigmatisation determined by the demographic variables (age, gender, and ethnicity)

When the question goes beyond those respondents’ personal experience to ask whether they know if their immediate social contacts have encountered racist comments and/or discrimination during the COVID-19 pandemic, ethnicity (*χ*^2^ = 34.081, df = 3, *p* = 0.000 < 0.05) becomes the only variable that has resulted in statistically significant differences in respondents’ answers. This can be also seen in Table 3, which shows that significantly more Chinese (58.2%) than Indians (23.2%), Filipinos (22.9%), and ‘other Asians’ (35.6%) answered ‘yes’ to this question. Vice versa, significantly less Chinese (41.8%) than the other three ethnic groups (Indians — 76.8%, Filipinos — 77.1%, and other Asians — 64.6%) answered ‘no’ to this question.

Ethnicity (*χ*^2^ = 80.212, df = 6, *p* = 0.000 < 0.05; *χ*^2^ = 86.490, df = 3, *p* = 0.000 < 0.05) is also an important variable which has contributed to the statistically significant differences in respondents’ answers about whether they have noticed any racist comment against their ethnicity in the media/social media and any stigmatisation associated with COVID-19 during the pandemic. Table 3 shows that significantly more Chinese (67.9%) than the other three Asian groups (Indians — 24.2%, Filipinos — 21.0%, and other Asians — 44.8%) perceived racist comments in the media and/or social media. There is also a significant difference between ‘other Asians’ and Indians regarding their perception of racist comments. In addition, significantly fewer Chinese (15.6%) did not notice any racist comments than the other three ethnic groups (Indians — 65%, Filipinos — 64.8%, and other Asians — 43.8%).

Similarly, Table 3 shows the fact that significantly more Chinese (85.7%) than the other three ethnic groups (Indians — 26.4%, Filipinos — 45.4%, and ‘other Asians’ — 45%) noticed stigmatisation associated with COVID-19 during the pandemic; and vice versa, significantly less Chinese (14.3%) did not notice stigmatisation compared with Indians (73.6%), Filipinos (54.6%), and ‘other Asians’ (55%).

Apart from ethnicity, age (*χ*^2^ = 14.410, df = 6, *p* = 0.025 < 0.05) and gender (*χ*^2^ = 6.777, df = 2, *p* = 0.034 < 0.05) are two variables which determine the statistical differences in respondents’ answers about whether they noticed any racist comment in media/social media because of COVID-19. Table 3 shows that significantly more respondents aged 50–64 years old (21.2%) than those aged 18–29 years old (8.0%) and significantly more women (17.1%) than men (9.4%) were ‘not sure’ whether they noticed any racist comment in the media/social media.

### The Correlation Analysis

As Table 4 shows, among those who experienced discrimination, 84.9% of respondents noticed stigmatisation of COVID-19 associated with China/Chinese/Asian-looking people, but 15.1% of the respondents did not notice any stigmatisation of COVID-19. Among those who did not experience discrimination, 39.6% of the respondents noticed stigmatisation because of COVID-19, while 60.4% of the respondents did not notice stigmatisation because of COVID-19. The *chi*-square test shows that the correlation coefficient is 0.402, indicating a moderate correlation [52]. Therefore, the conclusion is that the noticed stigmatisation because of COVID-19 and the perceived discrimination by the respondents are positively correlated.

**Table 4:** Correlation between the perceived racial discrimination and stigmatisation of COVID-19

		Have you noticed any stigmatisation associated with COVID-19 during the pandemic?	
		Yes	No
Since the COVID-19 outbreak in NZ, have you been discriminated against (e.g. making offensive remarks about your race, verbal/physical abuse) because of your ethnicity?	Yes	84.90%	15.10%
	No	39.60%	60.40%

Overall, the survey shows Asians experienced considerable racial discrimination related to the pandemic. In addition, ethnicity is a significant variable determining the differences in the level of discrimination that different Asian ethnic groups experienced and of the noticed stigmatisation of the virus. Despite the long history of discrimination against Chinese in the New Zealand society, in the COVID-19 pandemic context, the analysis results show that the Chinese also experienced pandemic-related racial discrimination and noticed the stigmatisation of the pandemic the most among all the Asian respondents. This situation is not surprising due to the general belief that China is the origin of the pandemic outbreak, and people of Chinese appearance are therefore likely picked up as the target for racist attacks and abuses and stigmatisation of the virus is often associated with China and Chinese people.

## Result 2: the Intersectionality of Stigmatisation and Discrimination, Health Governance, and Global Pandemic Politics — What Do the Interviews Reveal?

The interviews show that the factors contributing to Asian people’s sense of being discriminated against are various and associated with several social, cultural, and political elements. Firstly, the sense of being discriminated against is closely associated with the disparity of implementing the border control measures in New Zealand, as one of the Chinese interviewees discussed:

*It is strange [that] New Zealand banned travel from China before a case from there and banned Iran immediately after a case was found. However, New Zealand didn’t ban travel from Italy, USA, and Australia immediately after finding cases from those countries. I feel we Chinese are discriminated against and treated differently in this sense. You see, they discriminate against us; the consequence is: New Zealand’s early reluctance to implement equal border restrictions on many Western countries is the major reason for the arrival and spread of COVID-19 in this country.*

It has been discovered that at the onset of the COVID-19 outbreak, the New Zealand government implemented uneven travel bans for different countries [18]. For instance, travelling directly from China was banned on 3 February 2020 when there were no confirmed cases from China or via China. A similar decision in respect to the travel ban was made for Iraq. When the first infection case in New Zealand was confirmed from Iran on 28 February 2020, all travellers from Iraq were immediately banned from entering New Zealand on the same day. In contrast, after the first confirmed case from Italy was discovered in New Zealand on 4 March, the government did not ban travel from Italy until 11 March when nationwide lockdown was implemented by the Italian government. Similar border responses were enacted vis-a-vis many other Western countries during New Zealand’s early COVID-19 outbreak, such as the USA and Australia, which were major COVID-19 case sources for the virus’ development in New Zealand in the first year of the pandemic outbreak. It was not until 20 March 2020 that a full-scale border closure in New Zealand to all non-citizens and non-residents was enforced [26]. Another Indian interviewee discussed:

*Why just banned travels from China when there was no single case from there, but still allowed travels from Australia and USA where many infected cases from there at the beginning of the pandemic? Why were we Asians singled out and treated differently? I feel that we and Asian countries were perceived with racial bias in the COVID context. The pandemic just brought the issues of racial discrimination out of the surface and made it more obvious at the societal level. It is a prolonged issue though.*

What is underpinning this clear disparity in the implementation of New Zealand’s border control measures and these details of rolling out the country’s travel bans is largely political and ideological. New Zealand is an OECD country and one of the ‘Five Eyes’ alliance (i.e. an intelligence alliance comprising Australia, Canada, New Zealand, the UK, and the USA); this ideological position made the country reluctant to apply equal border control to all countries to eradicate COVID-19 at the onset of the pandemic. Cases showing a very similar politically driven pandemic response pattern can be found in many other places during the early stage of COVID-19 outbreaks, such as New York State in the USA [18]. Such a politically driven pandemic response has far more profound implications for the New Zealand Asian community. In particular, such an uneven implementation of the border controls invokes the Asian people’s sense of being racially discriminated against.

Secondly, the interviews find that Asian people’s sense of being discriminated against is closely related to the conspiracy theories associated with COVID-19. Taking a direct quotation from one of the Chinese interviewees as an illustration:

*I have a Kiwi colleague in our lab. One day, he commented that the US should ask for compensation from China because the virus started from there and probably is made from there. I educated him. I said: “You are a PhD in biomedicine, highly educated and knowledgeable. Based on your knowledge, can you tell me whether it is impossible to make the virus in a lab?” He replied, “No, it is almost impossible”. You see, rationally, he knows the truth, but he still made such terrible comments emotionally. I really cannot understand. It’s just unfortunate that the first outbreak of the pandemic happened in China. Why is the conspiracy theory getting so popular this time? I experienced SARs in 2003, and there was no such a thing, but why this time? I believe this is because some politicians want their political gain, and then they use the pandemic to blame a certain country. But who would suffer? It’s us, people from China.*

As we have witnessed, the pandemic has been stigmatised, associated with China, and used politically by some Western politicians, such as the previous president of the USA — Donald Trump, to stir up global anti-China sentiment [7]. What is behind the anti-China sentiment is the ongoing conflicts and controversial political disputes between China and the USA. The political tension between these two superpowers made the Trump administration try to grab any possible opportunity to suppress China, and conspiracy theories about the origin of the outbreak serve perfectly to advance this political agenda. Before the pandemic hit the world, the trade war between China and the USA, pro-democracy protests in Hong Kong, massive repression against Uighurs in Xinjiang, and increased Chinese and the USA naval activities in disputed waters off China’s coast had all served to heighten bilateral tensions [7]. The pandemic so far has proven to add a source of even greater friction. The finger-pointing and politically driven accusations and scapegoating between the world’s two leading powers have had catastrophic results to their conflicting diplomatic relations [10]. The public advocation of the conspiracy theories has not only worsened already tense relations between the USA and China but has also led to a public mood of hatred towards China and Chinese people, which has ‘spillover’ effects on other Asian groups [5] and makes members of other Asian subgroups, such as Vietnamese, Koreans, and Japanese, also becoming targets of anti-China/Chinese discrimination. It is no wonder that many Asian people feel discriminated against for their Asian ethnicities. This scenario can be evidenced by an interviewee who is originally from Philippines. He said:

*I found that among the most common posts about the COVID-related conspiracy theories on social media are those that describe the virus as “a biological weapon” made in a medical lab in China. You can imagine that when people firstly see that online, they would ask: “Is that true”? But when people see similar things several times, people would start to believe that “oh, it amybe true that the virus was manmade in a medical lab in China”. Consequently, all blames start to be towards China and people start to blame on Chinese-looking people. Saying bad things to them. As a Malaysian Chinese, I feel so vulnerable.*

Thirdly, the conspiracy theories about the origin of the outbreak go hand-in-hand with the stigmatisation of COVID-19. When the virus is referred to as the ‘China virus’ and ‘Chinese virus’, it hurts people’s feelings to who this refers to. One Chinese interviewee mentioned: ‘*It’s upsetting to hear the words “Chinese virus”. Why do they not call HIV an “American virus”? I am a New Zealand citizen, but I am also Chinese. When my ethnicity is racially mentioned and associated with this infectious disease, I feel this is so unfair*’.

The quote is not the only evidence found from the interviews that confirmed one of the findings from the online survey — that there is a positive correlation between the perceived stigmatisation of the pandemic associated with China and/or Chinese and racial discrimination, as another Chinese interviewee expressed:

*My second restaurant is in Browns Bay. You know that the majority of the customers are local kiwis. When New Zealand started to have the cases, I felt that the customers didn’t want to come anymore, even before the first lockdown. It was so obvious. Is this discrimination or just worrying about the virus? I don’t want to think more about it, but the fact is that the virus outbreak started in China, and this is a Chinese restaurant; therefore, they were suspicious towards us. “Oh, you are Chinese; maybe you have the virus”. They didn’t say so, but I could feel it. It’s subtle, and it’s hurting my feeling.*

These two quotations above confirm once again what the online survey has discovered — the perceived stigmatisation of the pandemic associated with China/Chinese/Asian-looking people is highly likely to lead to an increasing sense of being racially discriminated against for Asian people. It is worth pointing out that the disparaging rhetoric of the Trump administration that used the terms ‘Chinese virus’ and ‘China virus’ was indeed in their attempt to cover their incompetence in handling the outbreak in the USA and failure in global health leadership; and therefore, they tried to divert public attention on blaming others by scapegoating the Chinese and/or China [7, 10]. In China, on the other hand, the government’s rapid intervention to contain the virus’s spread, in conjunction with the USA-China diplomatic row, has solidified nationalism and anti-Americanism among its many citizens. However, the usage of the phrase ‘China virus’ has touched a raw nerve and elicited a nationalist backlash in China [12], as well as its emigrants in overseas.

Mainstream English-language media also frequently adopted the COVID-19-related rhetoric used by USA politicians. The racially stigmatising language was frequently used to describe the pandemic in the USA and other Western countries that have close alliances with the USA. The influence of the media is global and powerful in shaping the public view of COVID-19 [53]. For example, quite a few right-wing conservative media empires controlled by the Murdoch Media Giant in Australia contributed to the display of insensitive and racist terminology about the pandemic and Asian people. An article entitled ‘Chinese Virus Pandemonium’ made it to the front page of the Melbourne newspaper, the *Herald Sun* [54], while The *Daily Telegraph* [16] ran the headline ‘China kids stay home’ on the front page.

Furthermore, the interviews found that Asian people’s sense of being racially discriminated against is closely related to their observation that Asian people were treated and portrayed as the scapegoat for other countries’ failure to contain the pandemic outbreak. One interviewee mentioned:

*China had the first outbreak, which gave much more time to other countries to get ready to stop or contain the spreading of the virus, but they failed. Why? They also have advanced technologies, medical infrastructure and social welfare system. I think they hold bias towards China. They didn’t trust China’s information and medical advice, a developing country. Their prejudice and ignorance, or if I can say – their Eurocentric perspective made their failure. After they failed, they blamed China and us - Chinese people.*

In this discussion, Eurocentric perspectives were used to facilitate the interviewee’s articulation, reflecting the historical root of xenophobia running rampant during a transmission of a contagious disease. In such a historical context, coloured ethnic people are often framed and deemed responsible as the cause or origin of the diseases because they are inferior, unsanitary, and more likely to carry diseases [55].

Lastly, the interviews also discovered that different opinions towards mask-wearing, particularly at the inception of the pandemic, also contributed to some Asian people’s encounters of being racially discriminated in New Zealand, as one Malaysian interviewee shared:

*Before the government encouraged people to wear masks, I felt caught in a dilemma every time when I wore my mask. I wore my mask when I went to the supermarket to protect myself, but I worried how the local Kiwis saw me differently because, at that time, many Chinese and Asians started to wear face masks, but local Kiwis did not. Everybody knows that the first outbreak of the pandemic is in China. Although I am not Chinese, when I wear a mask, I might be identified as a Chinese. I feared the locals would blame me.*

The interviewee’s concern indicated a fear of being racially profiled and treated as a walking symbol of a disease in the pandemic. In other words, a simple and logical application of the Personal Protective Equipment (PPE) for transmissive diseases has been racialised. In Yashadhana et al.’s work, such situation is called ‘maskaphobia’ which refers to the fear and racist targeting of Chinese people wearing face masks in the context of COVID-19 [45]. Consequently, this racialised application of the PPE might hinder people from using this preventive method to protect themselves from being infected. It appears that the virus is much more effective at crossing national boundaries and different populations but understanding the virus and different risk management methods applied by different cultures and countries is much harder. On top of cultural barriers, it signals how irrational the purportedly rational policy-making process can be [56], remembering that whether face covering is an efficient PPE for COVID-19 was debated for a long time before the World Health Organisation made the recommendation of using masks. In New Zealand, face-covering only became mandatory in most public spaces during its recent Delta outbreak since August 2021 [57]. Understandably, different cultures have different cultural assumptions about wearing face masks. Still, when it becomes a racial issue and causes biased views and aggressive behaviour towards the racial ‘others’, one has to question its deeper roots of why some people need to blame others. In the COVID case, the racialised otherness is the Asian people.

The phenomenon of maskaphobia also reflects that Asian people in New Zealand have suffered internalised racism [44]. When fearing the application of a PPT has the potential to cause encountering racial discrimination, the interviewee started self-census to think whether he should or should not wear his mask, as another interviewee revealed:

*Should I wear my mask? I asked myself the same question every time when I exposed myself in public during the pandemic. I always looked around to see whether the number of people who wore masks were more than those who did not wear masks. If I saw that people with masks were the majority, I felt more comfortable wearing my mask.*

## Conclusion

Historically, pandemics can affect human society in various ways. They can result in a massive loss of human life, undermine a country’s economy, weaken its military power, and even change a war’s course [13]. The COVID-19 crisis has become the most serious public health crisis since the early twentieth century. It has proven to have the power to shape international relations, human social relations, and wealth re-distribution. New Zealand is no exception. This paper focuses on one type of impact of the pandemic on the human society of New Zealand — that is, people’s relations with each other in the sense of the pandemic as related to racial prejudice, bias, stigma, and racism.

The research shows that living in a world with COVID-19 has brought racism to the surface against Asians who live in New Zealand. The survey results align with other research which shows that Māori, Pacific, and Asian people were more likely than others to say they had experienced racial discrimination since the start of the COVID-19 outbreak [25, 58]. The pandemic is like a catalyst for the most recent outburst of the anti-Asian sentiment. The global scenario of stigmatising COVID-19 in relation to China and Chinese caused many Asian people’s sense of discomfort, insecurity, and being discriminated against. The survey firstly finds that the Asian population of New Zealand has suffered substantial racial discrimination during the pandemic, evidenced by 22.7% of surveyed Asian respondents reporting that they had experienced personal-level discrimination against their ethnicity. The survey secondly shows that ethnicity is a significant determinant in the Asian respondents’ answers to the questions related to pandemic-related racial discrimination and stigmatisation. While Chinese experienced racial discrimination the most among all surveyed Asian respondents, the qualitative interviews reveal that stigmatisation of COVID-19 and its related racial discrimination has ‘spillover’ effects on other Asian subgroups. This can be evidenced by the fact that many other non-Chinese Asian interviewees expressed their deep concern over using face masks. Thirdly, both the online survey and in-depth interviews find a strong correlation between the perceived stigmatisation of COVID-19 associated with China/Chinese and the personal, racial discrimination experienced by New Zealand’s Asian people, especially the Chinese. Finally, the research reveals that factors that contributed to the sense of being discriminated against are associated with several social, cultural, historical, and political factors. Some factors are more local, such as New Zealand’s uneven border control policy, but many are global, such as the global pandemic politics and foreign relations.

Overall, the paper uses the New Zealand case to reflect that the COVID-19 pandemic is not just a public health crisis; it also informs different and intersecting areas of cultural perceptions, medical discourses, race relations, and regional international health governance. It is hoped that the paper can raise public awareness of the need for a socially and culturally cohesive and consolidated approach to contain the pandemic and to promote positive public responses, attitudes, and behaviours; thus, to facilitate building up a comprehensive and robust public health defence system with cultural and social responsiveness at its foundation to deal with possible future pandemics in New Zealand. These issues are also relevant to preparing for and responding to future pandemics and other disasters and are consistent with framework laid out in the National Disaster Resilience Strategy [59] and linked to the UN Sendai Framework for Disaster Risk Reduction.

## Data Availability

Data can be made available as requested but for sharing with the public.

## Footnotes

1 Asian people in this research are defined as those adult Asian residents in New Zealand, including those who were born in Asian countries as well as born in New Zealand. They have already obtained New Zealand residency, permanent residency, or citizenship. Based on the definition used by the New Zealand Census, Asian countries include those East Asian countries (e.g. China, Taiwan, North Korea, South Korea, Japan, and Mongolia), Southeast Asian countries (e.g. Philippines, Vietnam, Brunei, Indonesia, Singapore, Malaysia, Thailand, Laos, Myanmar, Cambodia, Timor-Leste, and Andaman and Nicobar), and South Asian countries (e.g. Nepal, India, Sri Lanka, Bangladesh, Bhutan, and Pakistan). Those Western and Central Asian countries are not defined as Asian countries by the New Zealand Census’s.

## Code Availability

It is available.
